# Supplementary material for: Modeling glioblastoma heterogeneity as a dynamic network of cell states
Source: Mol Syst Biol. 2021 Sep 16;17(9):e10105. doi: 10.15252/msb.202010105 (PMC8444284; doi:10.15252/msb.202010105)
Supplement: Supplementary file 6 — Source Data for Figure 5 [file MSB-17-e10105-s004.zip › Figure5A_sourcedata/GSEA_3017/nosigdb_stateC.GseaPreranked.1622795002785/DOROSZKO_U3180MG_CLUSTER_3.html]

Details for gene set DOROSZKO\_U3180MG\_CLUSTER\_3[GSEA]

|  || Dataset | state13017 |
| Phenotype | NoPhenotypeAvailable |
| Upregulated in class | na\_pos |
| GeneSet | DOROSZKO\_U3180MG\_CLUSTER\_3 |
| Enrichment Score (ES) | 0.7859719 |
| Normalized Enrichment Score (NES) | 6.768703 |
| Nominal p-value | 0.0 |
| FDR q-value | 0.0 |
| FWER p-Value | 0.0 |
Table: GSEA Results Summary

  

Fig 1: Enrichment plot: DOROSZKO\_U3180MG\_CLUSTER\_3      
 Profile of the Running ES Score & Positions of GeneSet Members on the Rank Ordered List

  

| PROBE | GENE SYMBOL | GENE\_TITLE | RANK IN GENE LIST | RANK METRIC SCORE | RUNNING ES | CORE ENRICHMENT || 1 | ATAD2 |  |  | 7 | 0.649 | 0.0032 | Yes |
| 2 | FAM111B |  |  | 8 | 0.616 | 0.0196 | Yes |
| 3 | HIST1H4C |  |  | 9 | 0.603 | 0.0357 | Yes |
| 4 | CENPU |  |  | 10 | 0.588 | 0.0513 | Yes |
| 5 | HIST1H1D |  |  | 12 | 0.583 | 0.0648 | Yes |
| 6 | FAM111A |  |  | 13 | 0.576 | 0.0802 | Yes |
| 7 | EZH2 |  |  | 14 | 0.574 | 0.0955 | Yes |
| 8 | CCNE2 |  |  | 15 | 0.570 | 0.1106 | Yes |
| 9 | CLSPN |  |  | 17 | 0.561 | 0.1236 | Yes |
| 10 | C21orf58 |  |  | 18 | 0.559 | 0.1385 | Yes |
| 11 | RRM2 |  |  | 19 | 0.542 | 0.1529 | Yes |
| 12 | HMGB2 |  |  | 20 | 0.534 | 0.1671 | Yes |
| 13 | MCUB |  |  | 21 | 0.533 | 0.1813 | Yes |
| 14 | XRCC2 |  |  | 26 | 0.495 | 0.1864 | Yes |
| 15 | CHAF1A |  |  | 27 | 0.494 | 0.1996 | Yes |
| 16 | PCLAF |  |  | 29 | 0.484 | 0.2105 | Yes |
| 17 | UBE2T |  |  | 31 | 0.475 | 0.2211 | Yes |
| 18 | GMNN |  |  | 38 | 0.453 | 0.2211 | Yes |
| 19 | ORC6 |  |  | 39 | 0.450 | 0.2331 | Yes |
| 20 | MMS22L |  |  | 40 | 0.448 | 0.2450 | Yes |
| 21 | ESCO2 |  |  | 43 | 0.440 | 0.2527 | Yes |
| 22 | UHRF1 |  |  | 45 | 0.436 | 0.2623 | Yes |
| 23 | RAD51AP1 |  |  | 46 | 0.435 | 0.2739 | Yes |
| 24 | PCNA |  |  | 48 | 0.435 | 0.2835 | Yes |
| 25 | TMPO |  |  | 49 | 0.432 | 0.2950 | Yes |
| 26 | DTL |  |  | 50 | 0.430 | 0.3064 | Yes |
| 27 | HIST1H1E |  |  | 51 | 0.430 | 0.3179 | Yes |
| 28 | TOP2A |  |  | 52 | 0.426 | 0.3292 | Yes |
| 29 | SPC25 |  |  | 53 | 0.415 | 0.3403 | Yes |
| 30 | BRCA1 |  |  | 54 | 0.410 | 0.3512 | Yes |
| 31 | DHFR |  |  | 55 | 0.405 | 0.3620 | Yes |
| 32 | WDR76 |  |  | 56 | 0.403 | 0.3727 | Yes |
| 33 | TYMS |  |  | 60 | 0.390 | 0.3771 | Yes |
| 34 | RRM1 |  |  | 62 | 0.386 | 0.3854 | Yes |
| 35 | SMC2 |  |  | 63 | 0.386 | 0.3956 | Yes |
| 36 | E2F1 |  |  | 67 | 0.380 | 0.3997 | Yes |
| 37 | CSRP2 |  |  | 72 | 0.376 | 0.4017 | Yes |
| 38 | PSIP1 |  |  | 73 | 0.374 | 0.4117 | Yes |
| 39 | DEK |  |  | 74 | 0.373 | 0.4216 | Yes |
| 40 | POLD3 |  |  | 76 | 0.367 | 0.4294 | Yes |
| 41 | CENPH |  |  | 77 | 0.366 | 0.4391 | Yes |
| 42 | SMC4 |  |  | 78 | 0.365 | 0.4489 | Yes |
| 43 | ITGB3BP |  |  | 79 | 0.364 | 0.4585 | Yes |
| 44 | CDCA5 |  |  | 81 | 0.361 | 0.4661 | Yes |
| 45 | GINS2 |  |  | 83 | 0.360 | 0.4737 | Yes |
| 46 | KLHL23 |  |  | 84 | 0.359 | 0.4833 | Yes |
| 47 | BARD1 |  |  | 86 | 0.357 | 0.4908 | Yes |
| 48 | DUT |  |  | 87 | 0.356 | 0.5003 | Yes |
| 49 | MIS18BP1 |  |  | 89 | 0.355 | 0.5077 | Yes |
| 50 | CARHSP1 |  |  | 90 | 0.353 | 0.5171 | Yes |
| 51 | MCM7 |  |  | 91 | 0.352 | 0.5265 | Yes |
| 52 | USP1 |  |  | 92 | 0.352 | 0.5359 | Yes |
| 53 | ASRGL1 |  |  | 93 | 0.350 | 0.5452 | Yes |
| 54 | ATAD5 |  |  | 97 | 0.345 | 0.5483 | Yes |
| 55 | FEN1 |  |  | 98 | 0.342 | 0.5574 | Yes |
| 56 | NUSAP1 |  |  | 100 | 0.340 | 0.5645 | Yes |
| 57 | H1FX |  |  | 101 | 0.339 | 0.5735 | Yes |
| 58 | NCAPD3 |  |  | 103 | 0.334 | 0.5804 | Yes |
| 59 | MCM8 |  |  | 104 | 0.331 | 0.5892 | Yes |
| 60 | SLBP |  |  | 106 | 0.331 | 0.5960 | Yes |
| 61 | GINS4 |  |  | 112 | 0.325 | 0.5946 | Yes |
| 62 | MCM3 |  |  | 114 | 0.324 | 0.6012 | Yes |
| 63 | H2AFX |  |  | 115 | 0.322 | 0.6098 | Yes |
| 64 | HIRIP3 |  |  | 116 | 0.321 | 0.6184 | Yes |
| 65 | PSMC3IP |  |  | 118 | 0.319 | 0.6249 | Yes |
| 66 | TIMELESS |  |  | 119 | 0.319 | 0.6333 | Yes |
| 67 | CCDC34 |  |  | 120 | 0.318 | 0.6418 | Yes |
| 68 | HAT1 |  |  | 121 | 0.318 | 0.6503 | Yes |
| 69 | SMC1A |  |  | 125 | 0.312 | 0.6526 | Yes |
| 70 | DNMT1 |  |  | 127 | 0.311 | 0.6589 | Yes |
| 71 | FBXO5 |  |  | 130 | 0.308 | 0.6630 | Yes |
| 72 | KIF15 |  |  | 136 | 0.302 | 0.6610 | Yes |
| 73 | NCAPG |  |  | 137 | 0.301 | 0.6690 | Yes |
| 74 | RFC4 |  |  | 138 | 0.300 | 0.6770 | Yes |
| 75 | CENPK |  |  | 139 | 0.299 | 0.6850 | Yes |
| 76 | LMNB1 |  |  | 142 | 0.293 | 0.6887 | Yes |
| 77 | ASF1B |  |  | 143 | 0.291 | 0.6965 | Yes |
| 78 | HNRNPAB |  |  | 144 | 0.290 | 0.7042 | Yes |
| 79 | HPF1 |  |  | 147 | 0.289 | 0.7079 | Yes |
| 80 | SIVA1 |  |  | 151 | 0.286 | 0.7095 | Yes |
| 81 | SUPT16H |  |  | 153 | 0.286 | 0.7151 | Yes |
| 82 | RFC3 |  |  | 156 | 0.283 | 0.7186 | Yes |
| 83 | RNASEH2B |  |  | 158 | 0.282 | 0.7241 | Yes |
| 84 | PARP1 |  |  | 163 | 0.280 | 0.7235 | Yes |
| 85 | MYBL2 |  |  | 164 | 0.278 | 0.7309 | Yes |
| 86 | H2AFY |  |  | 165 | 0.278 | 0.7383 | Yes |
| 87 | VRK1 |  |  | 169 | 0.275 | 0.7396 | Yes |
| 88 | CIP2A |  |  | 170 | 0.272 | 0.7468 | Yes |
| 89 | HELLS |  |  | 171 | 0.271 | 0.7541 | Yes |
| 90 | C19orf48 |  |  | 173 | 0.271 | 0.7592 | Yes |
| 91 | MAD2L1 |  |  | 174 | 0.270 | 0.7664 | Yes |
| 92 | RBBP8 |  |  | 177 | 0.267 | 0.7695 | Yes |
| 93 | MSH2 |  |  | 180 | 0.265 | 0.7725 | Yes |
| 94 | NCAPG2 |  |  | 187 | 0.260 | 0.7674 | Yes |
| 95 | XRCC5 |  |  | 188 | 0.259 | 0.7743 | Yes |
| 96 | MCM4 |  |  | 189 | 0.259 | 0.7812 | Yes |
| 97 | CDK1 |  |  | 191 | 0.255 | 0.7860 | Yes |
| 98 | PGAM1 |  |  | 218 | -0.258 | 0.7405 | No |
| 99 | CLIC1 |  |  | 237 | -0.261 | 0.7113 | No |
| 100 | METRN |  |  | 430 | -0.340 | 0.3340 | No |
Table: GSEA details [plain text format]

  

Fig 2: DOROSZKO\_U3180MG\_CLUSTER\_3: Random ES distribution      
 Gene set null distribution of ES for **DOROSZKO\_U3180MG\_CLUSTER\_3**
